# Effect of the Algaecide Palmitoleic Acid on the Immune Function of the Bay Scallop *Argopecten irradians*

**DOI:** 10.3390/molecules21050610

**Published:** 2016-05-10

**Authors:** Cheng Chi, Sib Sankar Giri, Jin Woo Jun, Hyoun Joong Kim, Sang Guen Kim, Saekil Yun, Se Chang Park

**Affiliations:** Laboratory of Aquatic Biomedicine, College of Veterinary Medicine and Research Institute for Veterinary Science, Seoul National University, Seoul-151742, Korea; chicheng0421@126.com (C.C.); giribiotek@gmail.com (S.S.G.); advancewoo@hanmail.net (J.W.J.); hjoong1@nate.com (H.J.K.); imagine5180@gmail.com (S.G.K.); arseidon@naver.com (S.Y.)

**Keywords:** Algaecide, *Argopecten irradians*, immune response, palmitoleic acid, harmful algal blooms

## Abstract

Palmitoleic acid (PA), an algicidal compound, is used against the toxin producing dinofagelate *Alexandrium tamarense*, however, its impact on the edible bay scallop (*Argopecten irradians*) is still unclear. Therefore, we investigated the impacts of effective algicidal concentrations (20, 40, and 80 mg/L) of PA on immune responses in *A. irradians*. Various immune parameters including acid phosphatase (ACP) activity, superoxide dismutase (SOD), lysozyme, phagocytic activity, total protein, malondialdehyde (MDA) level, and reactive oxygen species (ROS) production and the expression of immune-related genes (*PrxV*, *CLT-6*, *MT*, and *BD*) were measured at 3, 6, 12, 24, and 48 h post-exposure (hpe) to PA. Lysozyme activity was lower in scallops at 12–48 hpe to 80 mg/L. SOD, ACP activity, ROS production, the total protein, and MDA level was higher at 12 to 48 hpe with different concentrations of PA. Phagocytic activity increased at 6–12 hpe to 40–80 mg/L of PA, but decreased at 24–48 hpe. The expressions of genes *PrxV*, *CLT-6*, *MT* and *BD* down-regulated at 3 hpe were observed, while differential expressions from 6–48 hpe with different concentrations of PA. The present study demonstrated that immersing *A. irradians* in PA at effective concentrations could result in differential effects on non-specific immune responses and expressions of immune-related genes.

## 1. Introduction

Since the 20th century, population explosion and the rapid development of agriculture and industry have been accompanied by an apparent global increase in the occurrence, area, and harm (both ecological and anthropogenic) caused by harmful algal blooms (HABs). As chemical approaches are considered potentially dangerous for aquatic life and create environmental hazards, biological agents, including protozoa, viruses, and macrophytes, bacteria (e.g., bacterial secondary metabolites), are now being considered as potential suppressors for controlling the outbreak of HABs [1,2,3]. Several studies also revealed the existence of certain bacteria capable of inhibiting or degrading algal blooms in marine environments [4,5]. Very few studies have focused on the isolation of algicidal compounds from these bacteria. *Alexandrium tamarense* as a harmful algal species in the marine environment that can produce paralytic shellfish toxin. This toxin has heavily affected shellfish resources leading to serious economic losses in the shellfish aquaculture industry, also causing human illness, and even death [1,6]. Recently, Li *et al.* reported that an algicidal bioactive compound–palmitoleic acid (PA)—secreted from *Vibrio* sp. BS02 was used for controlling *A. tamarense* in Xiamen Sea Area, China [1]. As an algaecide, it has a half maximal effective concentration (EC_50_) value of 40 mg/L until 48 h and suppresses algal growth at concentrations higher than 20 mg/L. PA almost completely inhibited algal growth at 80 mg/L. Lysis of *A. tamarense* cells was observed under light microscopy after exposure to PA, and it also can effectively inhibit the growth of other harmful algae such as *A. minutum*, *Asterionella japonica* and *Heterosigma akashiwo* [1], however, the potential impact of the algicidal compound PA, which is used for controlling a harmful algal blooms in aquaculture settings, on other wild or cultivated species remains unknown.

Suspension-feeding bivalves naturally ingest most microalgae, and, thus, are exposed to a variety of toxic components. The accumulation and persistence of toxicity in bivalves is species-dependent and varies according to the concentration of the bloom and rates of feeding and toxin elimination in the shellfish [7]. When a bivalve is exposed to a toxic or noxious particle, shell-valve closure and reduced filtration may constitute the first response and may serve to minimize contact with the soft tissues [4]. Scallops are a cosmopolitan family of bivalves, widely distributed in some water areas including the seaward end of glaciers, subtropical and temperate estuarine bays, and tropical shallow seas [8], where HABs frequently happen. Many scallop species are highly prized as a food source, and some are farmed by the aquaculture industry. In recent years, interest in scallop immunity has continued to increase due to their economic importance and their key position in animal phylogeny and evolution. Therefore, scallops are good candidates for immunological studies. Scallop aquaculture, with an annual output of about 1 million metric tons, is one of the most important sectors of China’s mariculture industry. Most of the production comes from two species: the local zhikong scallop (*Chlamys farreri*) and the bay scallop *Argopecten irradians* [9], which was successfully introduced to China from North America in 1983, and has become a main maricultured shellfish species of China. It has been utilized as a cultured human food source for many years and thereby considered as economically important species [6].

It is generally assumed that scallop, an invertebrate, lacks the complexity of an adaptive immune system, relying solely on innate immunity mediated by both cellular and humoral components [10]. Cellular immune reactions, including encapsulation and phagocytosis, are performed by different haemocytes, and humoral immune responses consist of reaction cascades for microbe recognition, signal transduction, and immune effector productions [11]. During phagocytosis, large amounts of reactive oxygen species (ROS) are generated to kill the internalized bacteria, which is important for invertebrate survival [12], but damages will occur when the generation of ROS is excessive [13]. Production of excessive ROS and other pro-oxidants damages unsaturated lipids, breaks DNA bonds, as well as proteins, amino acids and carbohydrates [14]. Malondialdehyde (MDA) levels represent membrane lipid peroxidation status and this substance is always is used as a marker for the extent of oxidative damage [14]. However, in scallops, several antioxidant enzymes, such as superoxide dismutase (SOD) which is considered to be the first and most important line of defence against ROS and protects tissues from oxidative damage [15], catalase and extra cellular glutathione peroxidase have been identified, and all of them are involved in the host response against microbe challenges and environmental stress [11]. In addition, some antimicrobial compounds in humoral immune responses, like lysosomal enzymes, acid phosphatase (ACP) also participate in the degradation of foreign proteins, carbohydrates and lipids [1]. Therefore, phagocytosis, ACP, and SOD are essential components in the understanding of the immune response of scallops [12]. Total protein also plays a significant role in the immune response, and the modification of total protein levels can provide information from a whole-scope viewpoint on the processes of protein synthesis, post-translation modifications, protein degradation, and the interaction between proteins or other molecules, in response to environmental stress in marine animals [16,17].

To our knowledge, the effect of the algaecide PA on the immune responses of bay scallop has not been reported. Therefore, the present study investigated the interaction between PA and scallop immune responses, aiming to reveal impacts and any potential risk of using the algaecide PA on the non-specific immune responses of the bay scallop (*A. irradians*). Healthy scallops were stimulated by immersion in graded levels of PA for short durations, and temporal profiles of immune parameters were assessed. Furthermore, we also studied the effect of PA on the expression of immune-related genes.

## 2. Results and Discussion

### 2.1. Non-Special Immune Responses

Nowadays, environmental risk assessment (ERA) is widely accepted and consists of four main steps: hazard identification, a dose-response assessment, an exposure assessment, and risk characterisation. In an earlier study, palmitoleic acid (PA) was shown to have a 48 h EC_50_ value of 40 mg/L against *A. tamarense*, suppressing algal growth at a concentration higher than 20 mg/L [1]. PA almost completely inhibited algal growth at 80 mg/L, and selectively inhibited the growth of other harmful algae. An effective immune response is essential for maintaining the health of an organism, and may subsequently affect growth, reproduction, and ultimately, survival [18,19]. Therefore, the present study investigated how exposure to effective algicidal concentrations of an algicidal compound–PA—alters non-specific immune responses and the expression of immune-related genes in *A. irradians*. The results showed that PA could modulate non-specific immune responses and the expression of a series of immune-related genes in the bay scallop haemolymph. Therefore, the present work contributes towards the first step in the ERA procedure through the identification of effects of the algaecide PA on the bay scallop. This is an essential part of establishing the risks associated with developing and using algaecides for controlling and inhibiting HABs.

**Figure 1:**
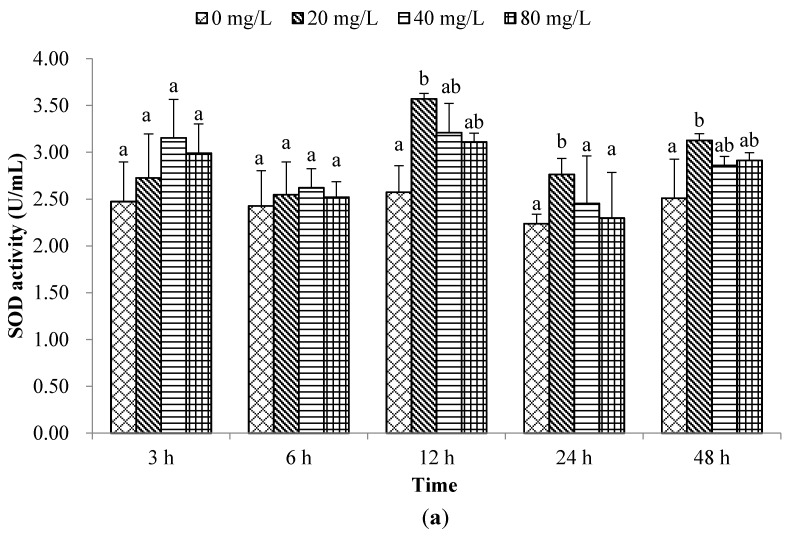

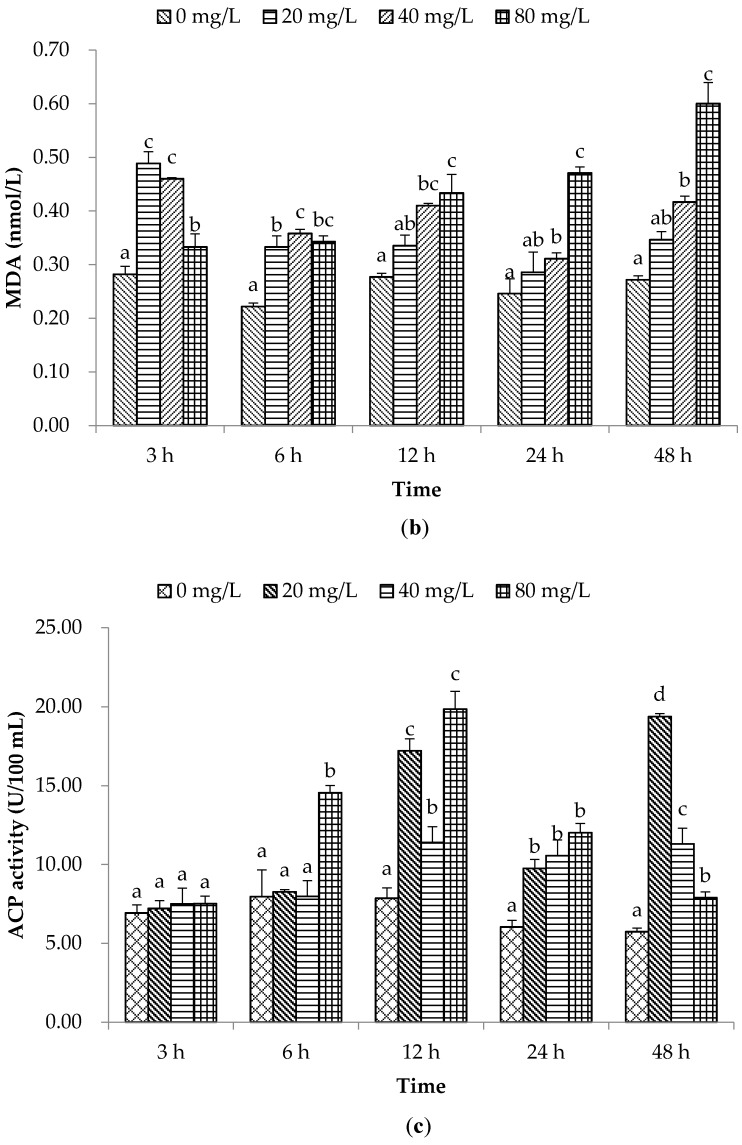

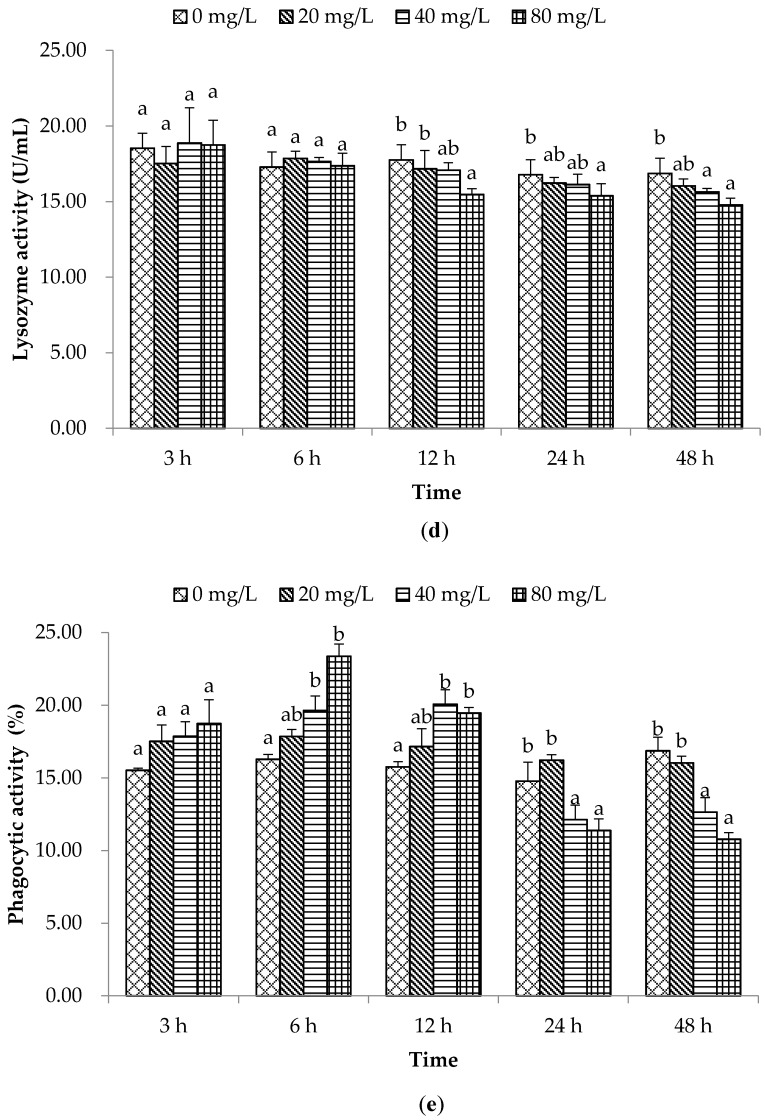

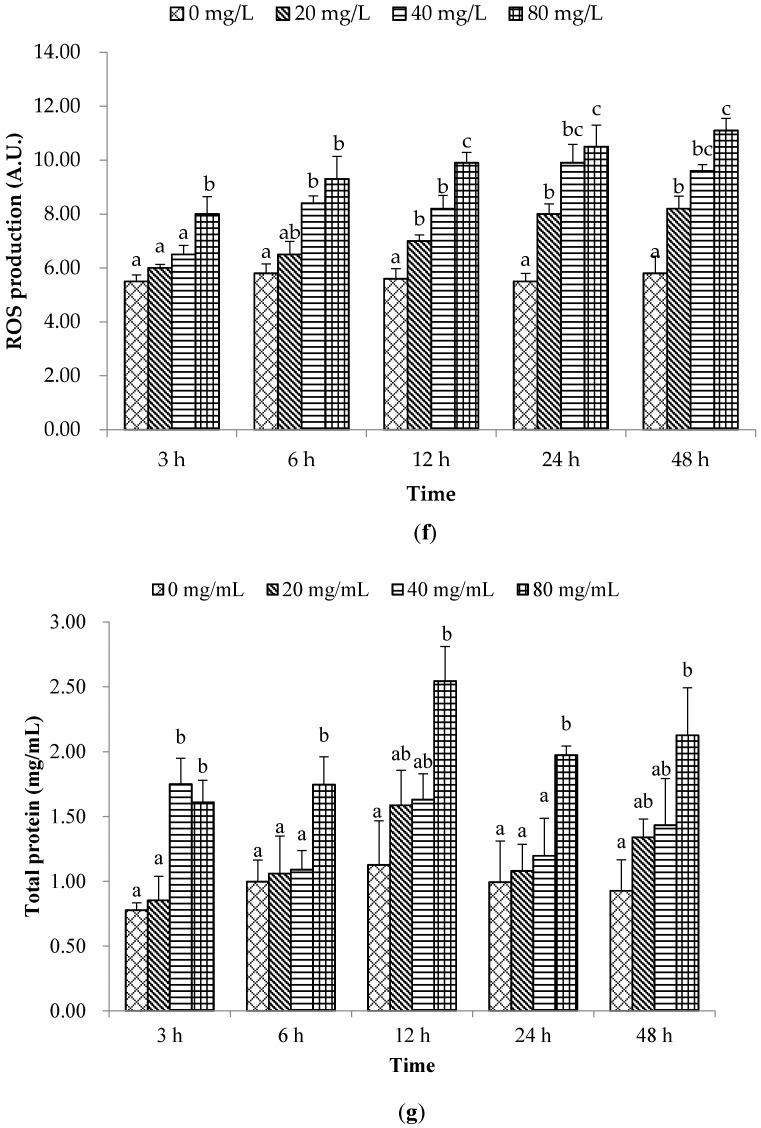
Effects of PA on non-special immune responses in bay scallop *A. irradians* at different time points after exposure to three concentrations (20, 40, and 80 mg/L). (**a**) SOD activity; (**b**) MDA content; (**c**) ACP activity; (**d**) lysozyme activity; (**e**) phagocytic activity; (**f**) ROS production (**g**) total protein; Data represent mean ± SD values (*n* = 3) at the same sampling time with different letters denoting significant differences (*p* < 0.05).

The rapid adaptation of a species to sudden changes in environmental oxygen content depends on its ability to increase its antioxidant production capacity [20]. Among these antioxidant systems, SOD is the first and most important defensive parameter [21]. SOD has been used as a biomarker for monitoring environmental pollution [22]. Malondialdehyde (MDA) is closely related to membrane lipid peroxidation status, therefore MDA content assay is always used to indirectly evaluate the extent of any oxidative damage [23]. In the present work, SOD activity was increased (*p* < 0.05) only at 20 mg/L of PA at 12, 24, and 48 h post-exposure (hpe) compared to control (Figure 1a). The MDA level at 3 and 6 hpe was significantly increased in all PA-treated groups, however from 12 to 48 hpe only in 40 and 80 mg/L treated groups (Figure 1b), which indicated that the haemolymph suffered from serious oxidative stress.

The current results were consistent with the earlier investigation that the MDA levels were significantly higher in *C. farreri* after exposure to ammonia-*N* [24]. Thus, as an indicator of oxidative damage, significantly higher MDA content in the 20 mg/L PA-exposed group at 3 and 6 hpe indicated that even a low concentration of PA could activate the antioxidant system and acute phase response system in scallops; however, it returned to a normal level at 12, 24 and 48 h with increasing SOD activity. On the contrary, the higher concentrations of PA suppressed the activation of SOD during the experimental period. Therefore, the higher MDA content in the 40–80 mg/L PA exposure groups may be due to the related lower SOD activity than at the lower concentration. The higher level of MDA even lead to peroxidation and cell damage, as it was considered to be a result of increased oxidative damage levels. Pan *et al.* [25] also reported a similar result whereby exposure to the chemical compounds benzo(α)pyrene and benzo(κ)fluoranthene induced SOD activity at low concentration, but this was delayed a certain time at higher concentrations with the increasing MDA level in the haemolymph of *C. ferrari*. These phenomena reflect that in the higher concentrations of PA groups, the related lower SOD activities could not eliminate the oxidative stress effectively, thus leading to increased MDA levels, and eventually leading to DNA damage or haemocyte apoptosis. Finally it seriously affected the immune function of bay scallops.

Acid phosphatase (ACP) is an important hydrolytic enzyme in phagocytic lysosomes sensitive to environmental stress [21]. In our work, although ACP activities at 6 hpe were higher (*p* < 0.05) only in the group which received 80 mg/L of PA, while at 12, 24, and 48 hpe they were higher (*p* < 0.05) in all PA treated groups than control (Figure 1c), showing that ACP activities in pre-stimulated scallops were significantly higher than those of un-stimulated scallops exposed to different concentrations of PA at various time intervals. Stronger ACP activity could enable the phagocyte to destroy and clear pathogens more effectively, conferring to scallops an increased resistance against long-term pathogen invasion. Our results are consistent with previous work reported by Jing *et al.* [22] who observed increased ACP activity in *Pinctada fucata* in response to copper exposure. In light of earlier reports, our results indicated that ACP activity was modulated to protect against PA immersion.

In the present study, lysozyme activity was lower (*p* < 0.05) in 80 mg/L of PA treatment group after 12, 24, and 48 hpe (Figure 1d). These results suggest that long time exposure to a high concentration of PA can reduce the lysozyme activity of scallops. Phagocytosis of haemocytes as well as the lysosomal enzyme levels usually act as important parameters to evaluate the immunotoxicity of environmental stimuli or pollutions to bivalves [13]. In the present study, the phagocytic activity was significantly different only at the two higher concentrations (40 and 80 mg/L of PA) with an increase at 6 and 12 hpe and a decrease at 24 and 48 hpe (Figure 1e), which suggested a negative relationship between the PA concentrations and exposure time. These results implied that a short duration PA exposure could stimulate phagocytosis of the haemocytes of scallops, but more than 24 h exposure with PA may inhibit the phagocytic activity even causing DNA damage and apoptosis due to the sequential accumulation of ROS. Therefore, our results suggested that lysozyme and phagocytic activity in the bay scallop are useful parameters for monitoring the potential impact of environmental hazards or aquatic toxins on bay scallop non-specific immunity.

Reactive oxygen species (ROS) production in the current investigation was increased (*p* < 0.05) at 12, 24, and 48 hpe with 20, 40 and 80 mg/L of PA, at 6 hpe with 40 and 80 mg/L of PA and at 3 hpe only with 80 mg/L of PA (Figure 1f). ROS production is another important mechanism of bivalve cellular immunity and most involves the superoxide ion radical O_2_^−^, hydrogen peroxide H_2_O_2_, and the hydroxyl radical OH [21]. Although a small amount of ROS is necessary to enhance the internal defences against pathogens, a series of damage will occur when the generation of ROS is excessive [13]. In this work, PA was found to enhance ROS production compared with the control group, and this was in line with the results shown by Liu *et al.* [13] in which a polychlorinated biphenyl isomer mixture (Aroclor1254) could increase ROS generation in the scallop *C. farreri*. In addition, the higher ROS production was observed at the higher PA concentrations as well as over the increased exposure time. These suggested that the higher concentration and longer PA exposure caused accumulation of reactive oxygen species (ROS) with stronger potentially toxic effects on bay scallops.

Tomanek [17] revealed that levels of a common set of stress-induced proteins including molecular chaperones that stabilize denaturing proteins increase during cellular stress. In the present work, the protein levels were higher (*p* < 0.05) in groups treated with 80 mg/L of PA at each time interval and with 40 mg/L at 3 hpe (Figure 1g). Our results were similar to those reported by Hannam *et al.* [19] where a significant enhancement of plasma protein concentration was observed in *C. islandica* following dispersed oil exposure. The current results may correlate with cytolysis in lysosome-enriched cells such as haemocytes, caused by high concentrations of PA.

### 2.2. Expression of Immune Related Genes

**Figure 2:**
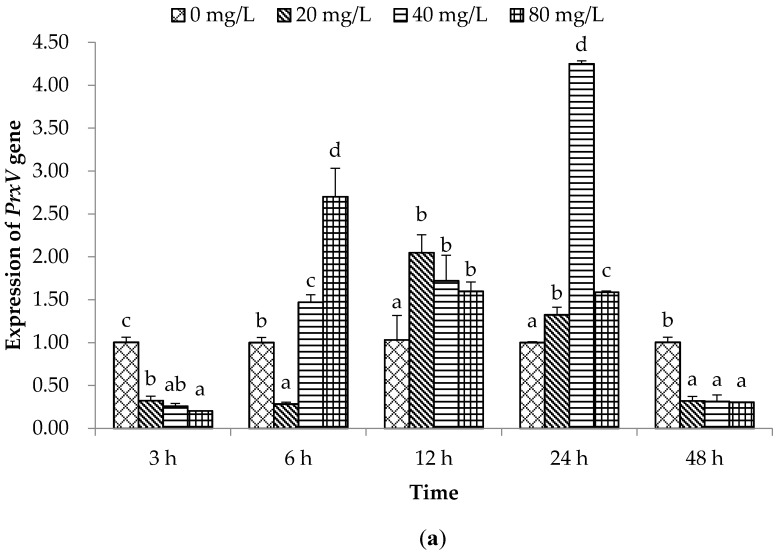

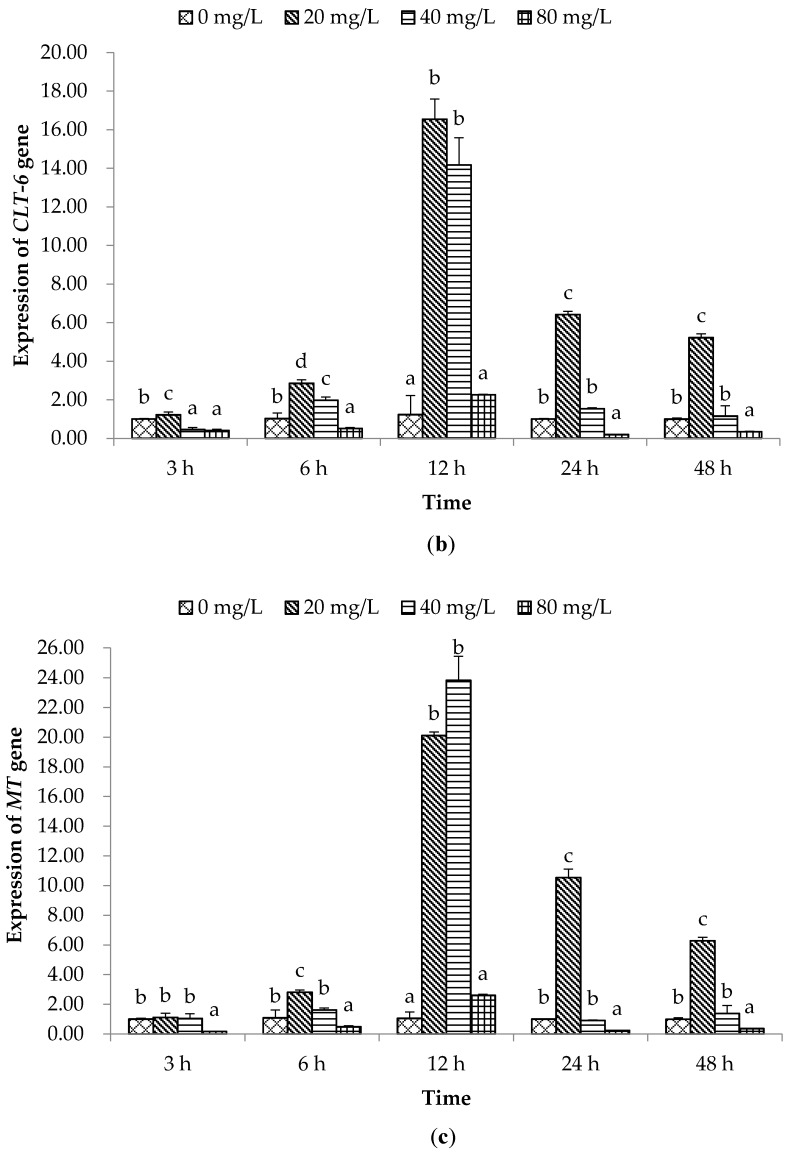

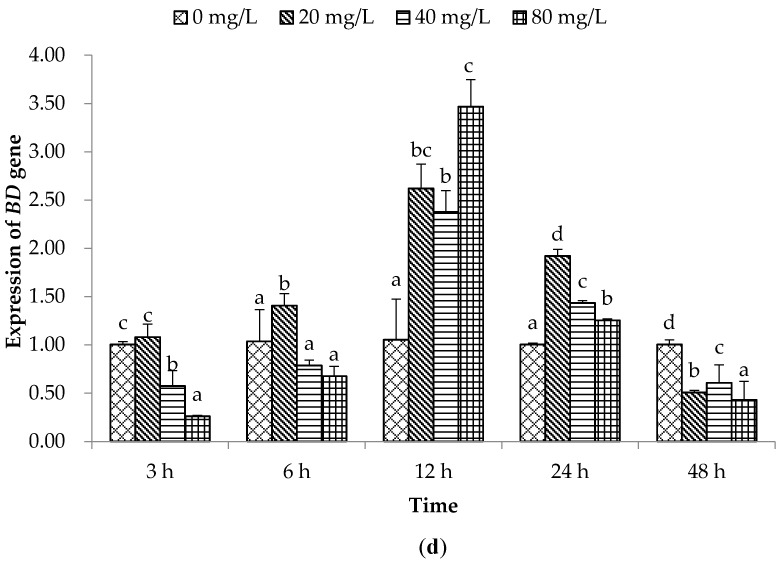
Effects of PA on immune related genes in bay scallop *A. irradians* at different time points after exposure to three concentrations (20, 40, and 80 mg/L). (**a**) *PrxV* gene; (**b**) *CLT-6* gene; (**c**) *MT* gene; (**d**) *BD* gene; Data represent mean ± SD values (*n* = 3) at the same sampling time with different letters denoting significant differences (*p* < 0.05).

Under normal conditions, the intracellular level of ROS is strictly maintained and controlled by peroxiredoxins (Prxs) and other enzymes. Prxs comprise a family of ubiquitously expressed proteins, such as 2-Cys (PrxI–IV), atypical 2-Cys (PrxV) and 1-Cys (PrxVI) [26]. PrxV is an indispensable part of an integrated cellular antioxidant defence network, which prevents ROS-mediated damage and ensures that cells respond appropriately to increasing levels of oxidative stress through H_2_O_2_-mediated signalling pathways [27]. Our results showed that *PrxV* expression was down-regulated (*p* < 0.05) at the initial and final hour of PA treatment; however, *PrxV* expression was higher (*p* < 0.05) in PA treatment groups at 6–24 h, except 6 hpe with 20 mg/L of PA, than in the control group, and declined rapidly at 48 hpe (Figure 2a). This phenomenon suggests that PA might be able to inhibit *PrxV* expression immediately following a short exposure time; although the higher concentration of PA may stimulate greater ROS production, which induces higher *PrxV* expression to protect against damage from ROS, as exposure time increasing it could inhibit the *PrxV*-mRNA expression significantly.

C-type lectins act as a first line of defence against pathogens. They can recognize and bind to terminal sugars on glycoproteins and glycolipids, and play significant roles in non-self-recognition and the clearance of foreign particles, either as cell surface receptors for microbial carbohydrates or as soluble proteins existing in scallop tissue fluids [28]. We found that scallops stimulated with 20 mg/L of PA had significantly higher (*p* < 0.05) *CLT-6* expression at each time interval; however exposure with 40 mg/L of PA resulted in higher *CLT-6* expression than control only at 6 and 12 hpe, and the highest expression was observed at 12 hpe. The 80 mg/L of PA dose attenuated (*p* < 0.05) the expected expression of *CLT-6* mRNA at 3, 6, 24, and 48 h (Figure 2b).

A previous study reported that *Listonella anguillarum* could induce significant up-regulation of the mRNA level of C-type lectins in scallop haemocytes [28]. This result suggested that *CTL-6* in bay scallop haemolymph could effectively be induced by a lower concentration of PA, and strong transcription of *CTL-6* was needed to synthesize and recruit proteins to defend against environmental stress and/or an invading pathogen; however, higher PA concentrations could inhibit *CLT-6* expression, which is a negative influence on immune responses of bay scallops.

Metallothionein (MT) is another group of molecules chiefly involved in the response to oxidative stress, especially from toxic metals [29]. MT induction has also been found to occur dramatically in response to tissue injury, infection and inflammation; therefore, MT is probably of great importance to the immune defence system of scallops [30,31]. In the present study, similar to the *CLT-6* gene expression, scallops treated with 20 mg/L of PA had a higher (*p* < 0.05) *MT* expression from 6–48 hpe and with 40 mg/L of PA only at 12 hpe than was seen in the control, and the highest expression was observed at 12 hpe (Figure 2c). Moreover, the highest concentration of PA (80 mg/L) attenuated (*p* < 0.05) the expected expression of *MT*-mRNA, except at 12 h. MT could protect cells from oxidative stress not only through metal binding/release dynamics, but also by acting as a scavenger of free radicals and reactive oxygen metabolites [31,32]. PA might be able to increase the disorder of oxidation/reduction reactions and provoke oxidative stress in scallops or induce an oxidative burst in scallop haemocytes. The increase of *MT* mRNA expression in scallops indicated that MT is inducible by immune infection and it activates protective defence mechanisms against the rise of ROS and possible oxidative stress produced by the invading pathogens; however, the high PA concentration (80 mg/L) could inhibit *MT* expression, and drop the ability of response to oxidative stress, especially from toxic metals.

Antimicrobial peptides (AMPs) are small cationic molecules widely distributed in all organisms, and they are thought to be a common feature of non-specific immunity in animals [33]. Big defensin (BD) is an AMP with remarkable microbicidal activity against Gram-positive and Gram-negative bacteria, and fungi in scallops [34]. In the present study, *BD* expression in haemolymph of scallops treated with 20 mg/L of PA was significantly higher (*p* < 0.05) at 6 to 24 hpe, and then decreased (*p* < 0.05) rapidly at 48 hpe. However, *BD* expression was higher at 12 and 24 hpe, but attenuated (*p* < 0.05) at 3, 6, and 48 h in groups treated with either 40 or 80 mg/L of PA (Figure 2d). This may be suggesting higher concentrations of PA suppressed *BD* mRNA transcription in the early stage of suddenly exposure to PA, and as time progressed, haemocytes were also mobilised to synthesise *BD* mRNA; perhaps other AMPs were expected to act as protector proteins during the late stage of this environmental stimulation. Moreover, the higher concentration may easily inhibit *BD* expression in the early stage of exposure, which restrained rapidly after short-term up-regulation during the late stage.

In general, the results of the present investigation demonstrated that exposure to different concentrations of PA modulates various immune parameters. Low concentration of PA had no significant effect on the immune parameters (SOD, ACP, phagocytic, lysozyme activities, total protein and ROS levels) at initial exposure time (at 3–6 hpe). However, at the highest concentration of PA, significantly higher activities in ACP, phagocytic, ROS, and total protein were observed at initial times point(s). Treatment with higher concentrations of PA for longer period (24–48 hpe) resulted in significant increment of ACP, ROS and total protein levels whereas the opposite result was seen for lysozyme and phagocytic activities. These results indicated that exposure to low concentrations of PA for shorter periods had less effect on the immune functions of bay scallop than the higher concentrations for longer time. Further, the MDA level was always higher in the treatment groups at the initial time points (3–6 hpe); however, at higher time points (12–48 hpe) increased (*p* < 0.05) level of MDA was only observed in the 40–80 mg·mL^−1^ treated groups. This result suggests that exposure to higher concentrations of PA for longer period caused more stress on the immune function of bay scallop. Our results suggest that the effect of PA on immune function of bay scallop is related to its dose and exposure time.

## 3. Materials and Methods

### 3.1. Palmitoleic Acid

Analytical-grade palmitoleic acid (PA) was obtained from Sigma-Aldrich Co. LLC (St. Louis, MO, USA) and stored at 4 °C in a refrigerator until use.

### 3.2. Animals

Bay scallops, *A. irradians*, averaging 60–70 mm in shell length, were collected from the Noryangjin fisheries wholesale market (Seoul, Korea) and maintained in lantern nets suspended in 800-L-capacity tanks containing filtered and aerated sea water to acclimatize to laboratory conditions (temperature: 10 ± 1 °C; salinity: 30 ± 0.1‰) for two weeks. Seawater was changed every day. Scallops were fed commercial shellfish diet (Instant Algae^®^ Shellfish Diet; Reed Mariculture Inc., Campbell, CA, USA) at a rate of approximately 1.2 × 10^10^ algae cells per scallop per day.

Bay scallops were randomly divided into a control group (without PA) and three treatment groups (with PA). There were three replicates for each treatment and control group. The treatment groups were treated with three concentrations (20, 40, and 80 mg/L) of PA that were reported as the minimum algicidal dose, EC_50_, and maximum effective algicidal dose against *A. tamarense*, respectively [1]. Three scallops from each replicate were randomly collected at 3, 6, 12, 24, and 48 h post-exposure (hpe). One mililiter of haemolymph was collected from adductor muscle using a 1-mL sterile syringe fitted with a 22-gauge needle within 1 min of removing a scallop from the tank. At each time point, an equal volume of haemolymph from three scallops in each replicate was pooled to reduce inter-individual variation. Individual scallops were sampled once to avoid repeated bleeding and/or handing stress. A 100-μL sample of haemolymph from each replicate was used for RNA extraction. Then, another 100-μL haemolymph from each replicate was centrifuged at 750× *g* for 3 min to collect the serum, which was then stored at −80 °C until determining humoral immune parameters. The rest of haemolymph maintained on ice until use for the measurement of phagocytic activity and reactive oxygen species production.

### 3.3. Measurement of Non-Specific Immune Responses

Superoxide dismutase (SOD) activity was determined according to the method described by Ōyanagui [35] using SOD kits (Nanjing Jiancheng Bioengineering Institute, Nanjing, China) following the manufacturer’s instructions. The optical density value (OD value) was measured at 550 nm. One unit of SOD was defined as the amount required to inhibit the rate of xanthine reduction by 50% in a 1-mL reaction system. Specific SOD activity was expressed as SOD units per mL of serum.

Malondialdehyde (MDA), a degradation product of lipid peroxidation known as thiobarbituric acid-reactive substance, was determined according to the thiobarbituric acid method using a MDA test kit (Nanjing Jiancheng Bioengineering Institute).

Acid phosphatase activity (ACP) in serum was spectrophotometrically measured using disodium phenyl phosphate as a substrate [36] using an acid phosphatase detection kit (Nanjing Jiancheng Bioengineering Institute). One unit of ACP activity was defined as the amount of enzyme in 100 mL of serum necessary to produce 1 mg of nitrophenol for 30 min at 37 °C.

Lysozyme activity was measured using lysozyme kits (Nanjing Jiancheng Bioengineering Institute) following the manufacturer’s instructions. One unit of lysozyme activity was defined as the amount of serum lysozyme that caused a decrease in absorbance by 0.001 per min at 530 nm.

The total protein concentration in scallop serum was determined using kits (Nanjing Jiancheng Bioengineering Institute) following the manufacturer’s instructions.

Phagocytic activity of phagocytic haemocytes was determined using the previously described method of Xue *et al.* [37]. Two hundred haemocytes were counted. Phagocytic activity, defined as the phagocytic rate (PR), was expressed as: PR = (phagocytic haemocytes/total haemocytes) × 100.

Reactive oxygen species (ROS) production was measured using reactive oxygen species kits (Nanjing Jiancheng Bioengineering Institute) following the manufacturer’s instructions. Fluorescence, quantitatively related to the ROS production of haemocytes without any stimulation, was measured at 500–530 nm by a fluorescence microplate reader. Fluorescence was expressed in arbitrary units (A.U.).

### 3.4. RNA Extraction and Reverse Transcription

Total RNA was extracted from haemolymph using TRIzol Reagent (CWBio, Beijing, China). The quality and purity of RNA was assessed by spectrophotometry, and the 260:280 ratios were 1.8–2.0. Afterwards, genomic DNA contamination was removed using DNase I (Promega, Madison, WI, USA). cDNA was synthesized using a PrimeScript™ RT Reagent Kit (TaKaRa Bio, Otsu, Japan) following the manufacturer’s instructions. The resulting cDNA was stored at −80 °C.

### 3.5. Real-Time Quantitative PCR Analyses of Gene Expression

The expression of immune-related genes, *PrxV*, *CLT-6*, *MT*, and *BD*, was carried out using real-time quantitative PCR (qPCR) (Qiagen, Hilden, Germany). All qPCR reactions were performed using SYBR Premix Ex Taq™ Perfect Real-Time Kits (TaKaRa Bio) and were conducted using a QiagenRotor-Gene Q RT-PCR Detection System (Qiagen). Gene expression was normalized using the housekeeping gene *β-actin*. PCR primer sequences used for qPCR are listed in Table 1 [1,28,31,34]. The reaction mixture included 10-μL SYBR Premix Ex Taq™, 1 μL of the forward and reverse primer (10 mM), and 1-μL cDNA. The remaining volume was filled with ultra-pure water to a final total volume of 20 μL. The reaction conditions and cycle index were conducted at 95 °C for 10 min followed by 40 cycles at 95 °C for 45 s, 56 °C for 45 s and 72 °C for 30 s. After the amplification phase, a melting curve analysis was conducted to eliminate the possibility of non-specific amplification or primer dimer formation. A standard curve was created from serial dilutions of sample cDNA. A standard curve was drawn by plotting the natural log of the threshold cycle (Ct) against the number of molecules. Standard curve of each gene was run in duplicate and three times for obtaining reliable amplification efficiency. The correlation coefficients (R^2^) of all standard curves were >0.99 and the amplification efficiency were between 90 and 110%. The relative expression ratios of the target gene in the treatment group *versus* those in the control group were calculated according to the following formula: Fold changes = 2 − ∆∆Ct, where ∆∆Ct = [Ct (treatment group) − Ct (treatment *β-actin*)] − [Ct (control group) − (control *β-actin*)] [38]. In all cases, each PCR was performed using three replicates.

### 3.6. Statistical Analysis

Normality and homogeneity of variance were tested using Kolmogrov–Smirnov and Cochran’s tests, respectively. All percentage data were arcsine-transformed, and the data were subjected to one-way ANOVA. Values are expressed as the arithmetic mean ± standard deviation (SD). Differences were determined using Duncan’s test in SPSS statistical software version 19.0 (IBM Corp., Armonk, NY, USA) with *p* < 0.05 indicating statistical significance.

## 4. Conclusions

The present study revealed an alteration in immune responses in bay scallop (*A. irradians*) caused by exposure to the investigated concentrations (20, 40, and 80 mg/L) of the algaecide PA. Although, it is not clear whether overall immunocompetence of the organism was compromised, exposure to different concentrations of PA can significantly affect several immune parameters and immune related genes of scallop. Therefore, these findings implicate a potential risk of popularizing and applying of PA as an algaecide management tool in scallop production. Also, the results of the present study may arouse researchers’ attention to investigate the impacts of the use of other algaecides to control algal bloom outbreaks in the marine environment. Currently, further studies are underway to explore the mechanism of action of PA. However, further work is required to assess the ability of *A. irradians* to respond to pathogen challenges following PA exposure to ascertain if PA can be tolerated by aquatic species, as the mollusc immune system consists of a multifaceted defence system, so suppression of one aspect of the immune function may be compensated for by another defence mechanism. 

## Figures and Tables

**Table 1:** Primers used for the analysis of mRNA expression by qRT-PCR.

Genes	Primer Sequence	Accession No.
*β-actin*	F: 5′CAAACAGCAGCCTCCTCGTCA 3′	AY335441
	R: 5′CTGGGCACCTGAACCTTTCGTT 3′	
*PrxV*	F: 5′AATCAAGGAGCGGCTGGCA 3′	HM461987
	R: 5′TCAACTTCTCAATCTTCCCGTCAT 3′	
*CTL-6*	F: 5′CAGTTGCTACAGGGTTCG 3′	GQ202279
	R: 5′GGGCGTTATCTGGCTCAT 3′	
*MT*	F: 5′AACTTGCTGTAGTGGGAATG 3′	EU734181
	R: 5′AGGCTGGAAACTGCTGTGGT 3′	
*BD*	F: 5′CGTGCCATACCCATTGCTTA 3′	DQ334340
	R: 5′ATGATTGTCGTTGCTCCTTGAT 3′	
